# Polymeric Materials: Surfaces, Interfaces and Bioapplications

**DOI:** 10.3390/ma12081312

**Published:** 2019-04-22

**Authors:** Alexandra Muñoz-Bonilla, Coro Echeverría, Águeda Sonseca, Marina P. Arrieta, Marta Fernández-García

**Affiliations:** 1 Instituto de Ciencia y Tecnología de Polímeros (ICTP-CSIC), C/Juan de la Cierva 3, 28006 Madrid, Spain; sbonilla@ictp.csic.es (A.M.-B.); cecheverria@ictp.csic.es (C.E.); agueda@ictp.csic.es (A.S.); 2 Interdisciplinary Platform for Sustainable Plastics towards a Circular Economy, SUSPLAST, 28006 Madrid, Spain; 3 Facultad de Ciencias Químicas, Universidad Complutense de Madrid (UCM), Av. Complutense s/n, Ciudad Universitaria, 28040 Madrid, Spain; marina.arrieta@gmail.com

**Keywords:** surface modification/functionalization, surface segregation, micro- and nanopatterned films, blends and (nano)composites, coatings, surface wettability, stimuli-responsive materials/smart surfaces, bioapplications

## Abstract

This special issue “Polymeric Materials: Surfaces, Interfaces and Bioapplications” was proposed to cover all the aspects related to recent innovations on surfaces, interfaces and bioapplications of polymeric materials. The collected articles show the advances in polymeric materials, which have tremendous applications in agricultural films, food packaging, dental restoration, antimicrobial systems and tissue engineering. We hope that readers will be able to enjoy highly relevant topics that are related to polymers. Therefore, we hope to prove that plastics can be a solution and not a problem.

Polymeric materials have moved from making the progress of the twentieth century to becoming the materials of the future to be reviled and persecuted by problems that were mainly generated by the ignorance of citizens, businesses and governments. These problems have resulted in the planet being contaminated and the resulting consequences. A world without plastics is hardly imaginable and for this reason, the European Community is proposing some goals related to the production, use and recyclability of plastics: (1) 60% reuse and recycling of all plastic packaging by 2030; and (2) 100% reuse, recycling and/or recovery of all plastic packaging in the whole EU by 2040 [1]. Recently, Devasahayam et al. [2] pointed out the advantages of recycling polymers in mineral and metallurgical processing. For example, plastics in e-wastes can be used as fuels and reductants in recovering valuable metals. In another example, the epoxy resins can be used as a binder/reductant or fuel source, which offers high compression strength under ambient conditions. This far exceeds the heat induration strength and provides savings in terms of costs, energy and emissions during the iron ore pelletization. Moreover, several review have focused on solid plastic waste recycling, discussing both mechanical and chemical recycling [3,4]. Of all types of waste, the largest amount of waste produced is packaging waste (near 40%), which has short life times. Therefore, EU has placed a limitation on single-use plastic to decrease this ratio. Another alternative to the recycling process and reducing the production of plastics from non-renewable resources is the use of biodegradable and/or bio-based polymers, respectively. 

One of the reviews in this special issue (SI) focuses on the use of natural and bio-based polymers as antimicrobial systems and their potential mainly in biomedical and food applications, but also in water purification and coating technology [5]. However, natural and bio-based materials frequently have lower performance than traditional synthetic polymers. Therefore, it is necessary to make modifications or adjustments during the processing steps in order to modulate their final performances. In one of the SI articles, Samper et al. [6] analyzed the influence of small amounts of biodegradable polymers, such as poly(lactic acid), polyhydroxybutyrate and thermoplastic starch in the recycled polypropylene. It is shown that the recycling of polypropylene blended with these bio-based and biodegradable polymers is hardly affected when it is used at a proportion higher than 5 wt %. In this sense, the review of Luzi et al. [7] presents the blending of bio-based and/or biodegradable polymers with traditional synthetic polymers for packaging applications with an optional use of bio-based nanofillers. This nicely highlighted how these bio-based materials enhance the gas/water/light barrier properties and the compostability and migration performance of blends. Moreover, they also discuss the effect of incorporating bio-based nanofillers on the overall behavior of nanocomposite systems that is constituted of synthetic polymers, which is combined with biodegradable and/or bio-based plastics. 

The use of natural polymers is also presented in another article wherein alginate crosslinking by CaCl_2_ is obtained to create modified-release drug delivery systems with mucoadhesive properties [8]. The authors present the production of microparticles by the spray drying technique, which enables us to obtain microparticles with a low moisture content, high drug loading, a high production yield and a prolonged release of soluble drugs. Peng’s group [9] reported the use of chitosan with wood auto-hydrolysates that are obtained in the pulping process by hydrothermal extraction, which contains a considerable amount of hemicelluloses and slight lignin, in order to form films by the casting method. These films possess a higher tensile strength, better thermal stability, higher transmittances, lower water vapor permeability and superior oxygen barrier properties compared to those without chitosan due to the crosslinking interaction between the components, which occurs due to the Millard reaction. In another article, Ma et al. [10] used fibers from waste corn stalks as reinforcing materials in friction composites. They found that the incorporation of corn stalk fibers had a positive effect on the friction coefficients and wear rates of friction composites. The results revealed that the satisfactory wear resistance performances of these materials are associated with their worn surface morphologies and the formation of secondary contact plateaus.

Moreover, another polysaccharide, chitosan, is applied for scaffold preparation in tissue engineering. In more detail, Francolini et al. [11] analyzed the conjugation of chitosan with graphene oxide. Depending on its oxidation degree, the resulting scaffolds present improved or reduced mechanical performance and best or worst cytocompatibility as tested in human primary dermal fibroblasts. Another review of Foster’s group [12] meticulously displays the problem of disc degeneration, which affects a great part of population, by describing the anatomy of the spine, the functions and biological aspects of the intervertebral discs. They point out that although there are numerous studies focusing on tissue engineering for disc degeneration, more progress needs to be made. 

Focusing on some actual problems, dental restoration failures remain a major challenge in dentistry. In another review, Xu’s group [13] provided information on the development and properties of innovative antibacterial dental polymeric composites, antibacterial bonding agents, bioactive root caries composites, adhesives and antibacterial and protein-repellent endodontic sealers. These polymeric materials substantially inhibit biofilm growth and greatly reduce acid production and polysaccharide synthesis of biofilms. Following with antimicrobial polymeric materials, Lienkamp’s group [14] describes the development of amphiphilic copolymers of oxanorbornene monomer bearing N-*tert*-butyloxycarbonyl protected cationic groups with an oxanorbornene-functionalized poly(ethylene glycol) macromonomer. After this, these comb-like copolymers are surface-attached to polymer hydrogels, giving rise to a material that is simultaneously antimicrobial and protein-repellent. In another article, Ji et al. [15] analyzed the antifouling behavior directly in the natural seawater of different carbon nanotubes-modified polydimethylsiloxane nanocomposites by using the multidimensional scale analyses method.

Palza et al. [16] carefully reviewed the development of polymeric materials with electroactivity, such as intrinsically electric conductive polymers, percolated electric conductive composites and ionic conductive hydrogels. They evaluated their use in the electrical stimulation of cells, drug delivery, artificial muscles and as antimicrobial materials.

On the other hand, the breath figures approach is presented as an efficient method to obtain highly ordered porous materials with potential applications in cell culture and antimicrobial coatings, respectively [17,18]. These articles discuss the influence of the chemical nature of polymers, the solvent or the humidity in the preparation process on the final properties (porous size, surface energy, etc.). Another approach is presented in the article of Lavieja et al. [19], where the use of a green laser in the range of nanosecond pulses was an effective method to obtain superhydrophobic and superhydrophilic surfaces on a white commercial acrylonitrile-butadiene-styrene copolymer and therefore, to control its wettability. The last article deals with the surface modification method to produce gradient wrinkles using a gradient light field. Li et al. [20] described the easy control of the gradient wavelength of wrinkles by modulating the distance between the lamp and the substrate.

Finally, we would like to thank all authors for contributing to this collection in “Polymeric Materials: Surfaces, Interfaces and Bioapplications”.
